# Apoptin Overexpression Efficiently Amplified Cytotoxic Effects of PI3K Inhibition Using BKM120 in Lymphoblastic Leukemia Cell Lines

**DOI:** 10.34172/apb.2022.064

**Published:** 2021-07-10

**Authors:** Ali Anjam-Najmedini, Rohollah Vahabpour, Ava Safaroghli-Azar, Alireza Kazemi, Parvaneh Movahhed, Majid Momeny, Davood Bashash

**Affiliations:** ^1^Department of Hematology and Blood Banking, School of Allied Medical Sciences, Shahid Beheshti University of Medical Sciences, Tehran, Iran.; ^2^Department of Medical Laboratory Sciences, School of Allied Medical Sciences, Shahid Beheshti University of Medical Sciences, Tehran, Iran.; ^3^Turku Centre for Biotechnology, University of Turku and Åbo Akademi University, Turku, Finland.

**Keywords:** Apoptin, Acute lymphoblastic leukemia (ALL), Gene transduction, PI3K signaling pathway, BKM120

## Abstract

***Purpose:*** Although the complex structure of acute lymphoblastic leukemia (ALL) and involvement of diverse pathways in its pathogenesis have put an obstacle in the way of efficient treatments, identification of strategies to manipulate the genome of neoplastic cells has made the treatment prospective more optimistic.

***Methods:*** To evaluate whether the transduction of apoptin __a gene encoding a protein that participates in the induction of apoptosis__ could reduce the survival of leukemic cells, we generated recombinant lentivirus expressing apoptin, and then, MTT assay, flow cytometric analysis of DNA content, western blotting, and quantitative reverse transcription polymerase chain reaction (qRT-PCR) were applied.

***Results:*** Transduction of apoptin into different leukemic cells was coupled with the reduction in the viability and proliferative capacity of the cells. Among all tested cell lines, Nalm-6 and C8166 were more sensitive to the anti-leukemic property of apoptin. Moreover, we found that the transduction of apoptin in the indicated cell lines not only induced G2/M cell cycle arrest but also induced apoptotic cell death by altering the balance between pro- and anti-apoptotic target genes. The efficacy of apoptin transduction was not limited to these findings, as we reported for the first time that the overexpression of this gene could potentiate the anti-leukemic property of pan PI3K inhibitor BKM120.

***Conclusion:*** The results of this study showed that the transduction of apoptin into lymphoblastic leukemia cell lines induced cytotoxic effects and enhanced therapeutic value of PI3K inhibition; however, further investigations are demanded to ascertain the safety and the efficacy of apoptin transduction in patients with ALL.

## Introduction

Despite the impressive advances in the improvement of leukemia management by the entrance of the wide range of novel anti-leukemic agents, a considerable proportion of patients suffering from acute lymphoblastic leukemia (ALL) still lose their lives due to the disease recurrence and treatment failure.^1^ This challenge provides an opportunity for allogeneic bone marrow transplantation to be considered as the sole solution for this failure, especially for pediatric patients.^2^ However, the successful outcome of this approach is profoundly restricted by the shortage of HLA-compatible donors, and complications, such as graft versus host disease, which in turn replaced this valuable therapeutic tactic with an alternative approach, autologous BMT.^3^ For an era, the advent of autologous bone marrow transplantation (BMT) has been assumed to be a beneficial strategy for the treatment of human leukemia; however, the inefficient purging of leukemic cells from the autograft has put another obstacle in the way of successful BMT.^4^ Given these, it is not surprising that ever-increasing attempts are established not only to increase the efficacy of this treatment protocol but also to open the window of total remission for leukemic patients.

Gene therapy has long been considered to be the sole answer to many unsolved medical problems. One of the applications of this technique in cancer treatment could be mediated through integrating a specific gene into the genome of neoplastic cells and forced apoptotic cell death.^5^ Among a wide variety of the genes that could be incorporated with the leukemic genome, apoptin, a small protein encoded by chicken anemia virus, is one of the most studied ones.^6^ What centralized apoptin in the recent cancer investigations is due to its unique property to induce apoptotic cell death in malignant cells, but not in normal counterparts.^6-8^ Notably, this specific characteristic has been suggested to be associated with the phosphorylation status of the proteins, as in malignant cells the phosphorylated apoptin translocates to the nucleus and plays an essential role in apoptosis.^6^ However, in normal cells, this protein remained un-phosphorylated and be susceptible to the proteasome-dependent degradation.^8^ This remarkable property encouraged us to evaluate the anti-leukemic activity of apoptin in leukemic cells and assessed whether its transduction could recruit the induction of apoptotic cell death in leukemic cells.

## Material and Methods

### Cell lines

HEK 293T, Nalm-6 and Reh (human pre-B ALL cell lines), C8166 (Adult T-cell leukemia/lymphoma), and KMM-1 (multiple myeloma cell line) cells were cultured according to the National Cell Bank of Iran, Pasteur Institute, Iran. Briefly, cells were cultured in RPMI 1640 medium (Biosera, France) supplemented with 10% heat-inactivated fetal bovine serum, 100 U/mL penicillin and 100 g/mL streptomycin in a humidified 5% CO_2_ atmosphere at 37°C under standard cell culture conditions.

### Generation of recombinant lentiviral vector

The full-length coding sequence of apoptin (chicken anemia virus VP3 gene) was commercially synthesized (Biomatika, Canada). pUC57 containing apoptin coding sequence was double digested and sub-cloned in the similarly double digested *pCDH*-*CMV*-*GFP*-*T2A*-*Puro* (System Biosciences, Palo Alto, CA) downstream of the CMV promoter. To produce recombinant lentivirus, HEK 293T cells were co-transfected with *pCDH*-*CMV*-*GFP-apoptin*, pSPAX2 plasmid (gag/pol-encoding plasmid), and pMD2.G (VSV-G envelope-encoding plasmid) using Lipofectamine^TM^ 3000 Reagent (Thermo Fisher Scientific). The supernatant was collected at 48 and 72 hours after transfection and then centrifuged at 48 000 g at 4°C for 4 hours. The cell pellets gently re-suspended in RPMI-1640 medium.

### Transduction of target cells

Cell lines were separately transduced with recombinant lentivirus expressing apoptin (LV-GFP-apoptin) and control lentivirus (LV-GFP) with only GFP expression. Briefly, 5×10^5^ cells were cultured in 500 µL of growth medium in a 24-well plate. Subsequently, 500 µL of concentrated LV-GFP particles containing 2.5 µg/mL polybrene (Merck, US) was added to each well (MOI = 50) and centrifuged at 1400 g for one hour to increase the transduction efficiency. After spinning, the transduced cells were incubated for 4 hours in a CO_2_ incubator, and then 500 µL of the medium replaced with a fresh complete growth medium.

### MTT assay

Inhibitory effects of apoptin expression on the metabolic activity of cell lines were determined using the methylthiazolyldiphenyl-tetrazolium bromide (MTT) assay. For this purpose, transduced leukemia cells were seeded in a 96-well culture plate (5×10^3^ cells/well/ 100 µL) and incubated with predetermined concentrations of BKM120 (2 µM) in combination. After incubation, treated and untreated groups were further incubated with MTT solution (5 mg/mL in PBS) in each well, and the plates were incubated in the dark at 37°C for 2–4 hours. After medium removal, the formazan product was solubilized by adding dimethyl sulfoxide and the optical density was measured at 570 nm in the ELISA reader.

### Trypan blue exclusion assay

Trypan blue exclusion assay was carried out to determine the antiproliferative effects induced by apoptin in leukemia cells as described earlier.^11^ Briefly, transduced leukemia cells were re-suspended in equal volumes of medium and trypan blue solution (0.4%) (Invitrogen). Then, the enumeration of viable cells was assessed using a hemocytometer chamber. Finally, the cell viability index was assessed as follows: viability (%) = viable cell count/total cell count × 100.

### Flow cytometric analysis of DNA content

For detection of DNA content and evaluation of the sub-G1 fraction that mainly represents dead cells, we used propidium iodide (PI) staining of inhibitor-treated cells. Briefly, cells were seeded into six-well plates at the concentration of 1 × 10^6^ cells/well and incubated with apoptin and BKM120-plus-apoptin. Cells were then harvested, washed twice with PBS and fixed with 70% ethanol at -20°C overnight. Afterward, cells were treated with RNase in PBS and incubated at 37°C before staining with PI for 30 minutes. Finally, cellular DNA content was quantified from the peak analysis of flow cytometric DNA histograms.

### Western blotting

Cells were centrifuged 48 h after treatments and cellular pellets were washed with cold PBS and lysed in RIPA buffer containing protease and phosphatase inhibitor cocktails (Sigma). After determination of protein concentrations according to the Bradford method, equivalent amounts of total cellular proteins were separated by 10% SDS-PAGE, and subsequently transferred to nitrocellulose membrane using a semidry transfer cell (Bio-Rad). The proteins were detected using specific primary antibodies and the enhanced chemiluminescence detection system according to the manufacturer’s protocol.

### Quantitative reverse transcription-PCR (qRT-PCR)

Total RNA was isolated by using the High Pure RNA Isolation Kit according to the manufacturer’s recommendation (Roche). The quantity of RNA samples was assessed spectrophotometrically using Nanodrop ND-1000 (Nanodrop Technologies, Wilmington, DE). Reverse transcription (RT)-reaction was performed using the RevertAid First Strand cDNA Synthesis kit (Takara BIO) according to the manufacture protocol. Real-time PCR was performed on a light cycler instrument (Roche Diagnostic, Manheim, Germany) using 10 μL of SYBR Premix Ex Taq technology (Takara BIO, Japan), 2 μL of cDNA product, 0.5 μL of each forward and reverse primers (10 pmol), and 7 μL of nuclease-free water in a total volume of 20 μL. Thermal cycling conditions included an initial activation step for 30 seconds at 95°C followed by 40 cycles including a denaturation step for 5 seconds at 95°C and a combined annealing/extension step for 20 seconds at 60°C. A melting curve analysis was applied to verify the specificity of the products, and the values for the relative quantification were calculated based on 2^-∆∆Ct^ relative expression formula: ΔΔC_T_ = (C_T Target_ – C_T ABL_) experimental sample – (C_T Target_ – C_T ABL_) control samples, where C_T_ is the cycle threshold.

### Statistical analysis

The data are expressed by mean ± standard deviation (SD) of three independent assays, each done in triplicate. An independent test was performed for comparison between the groups. Statistical significance was calculated using paired two-tailed Student’s *t*tests. Statistically different values were defined as significant at *P* < 0.05.

## Results

### Transduction efficiency of the constructed lentiviral vectors and verification of apoptin expression in leukemic cells

**Figure 1:**
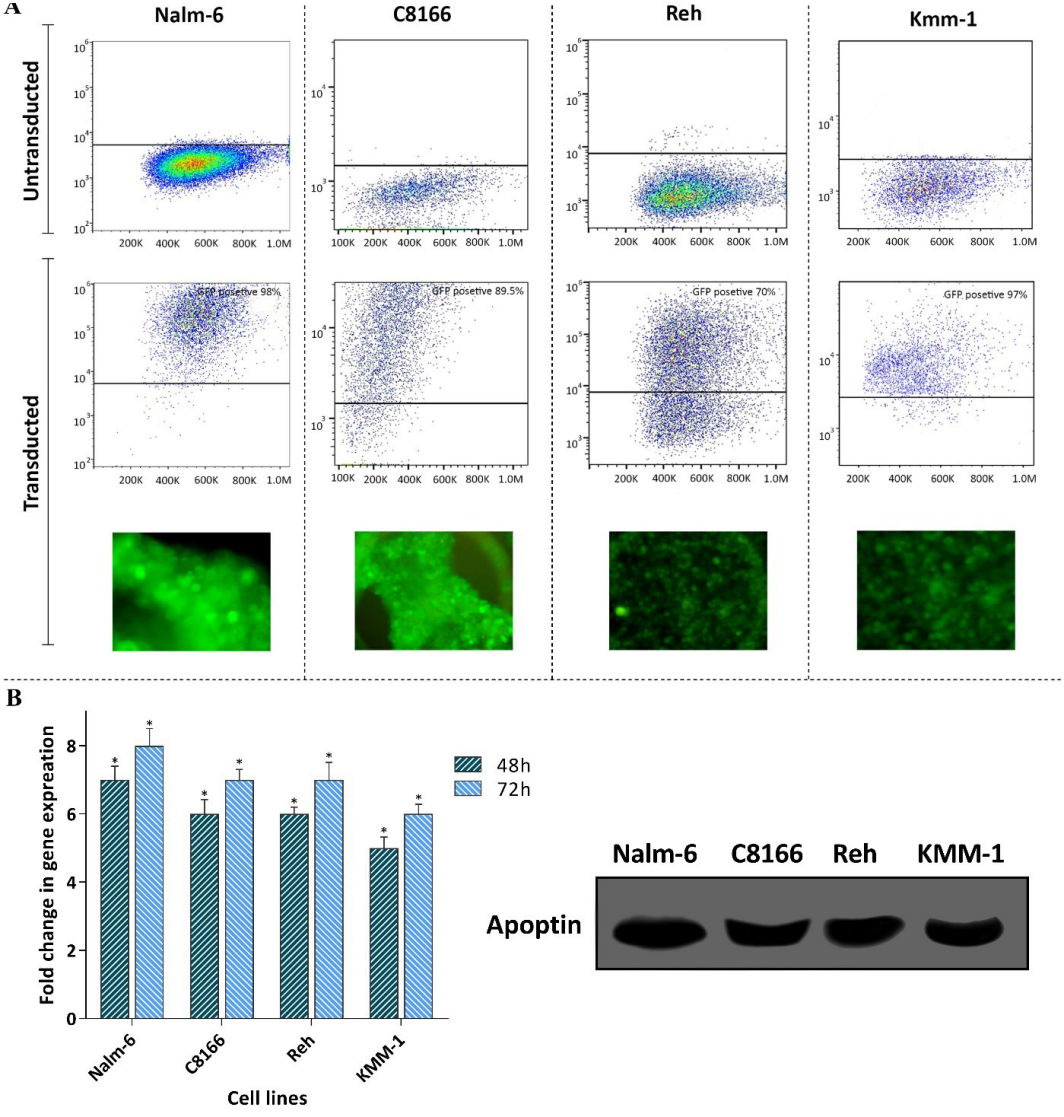
Evaluating the transduction efficiency and the expression of apoptin in a panel of leukemic cell lines. **A)** The transduction efficiency was evaluated using both flow cytometry and fluorescence microscopy.** B)** The results of both qRT-PCR and western blot analysis showed the expression of apoptin in the cell lines. Values are given as mean ± S.D. of three independent experiments. * *P* ≤ 0.05 represented significant changes from the control.

To test the anti-tumor property of apoptin gene on hematologic malignancies, we first constructed lentiviral vectors (LV-GFP and LV-GFP-apoptin), and then a panel of leukemic cell lines with different origins was chosen to be subjected to the indicated vectors (MOI = 10). The transduction efficiency was evaluated using both flow cytometry and fluorescence microscopy. As presented in Figure 1A, the resulting data declared that a significant population of the tested cells were positive for GFP. Moreover, the capability of the LV-GFP-apoptin vector in the expression of the gene was also studied using qRT-PCR and Western blot analysis in these cells. The results of qRT-PCR analysis clearly delineated that as a result of apoptin transduction, each cell line found the ability to express this gene (Figure 1B). In agreement with the results of qRT-PCR analysis, immunoblot analysis of the lysates of transduced cells showed the expression of apoptin protein with the size of approximately 40 kDa (Figure 1B).

### The expression of apoptin in leukemic cells was coupled with the reduction of cell survival through regulation of apoptosis-related genes

**Figure 2:**
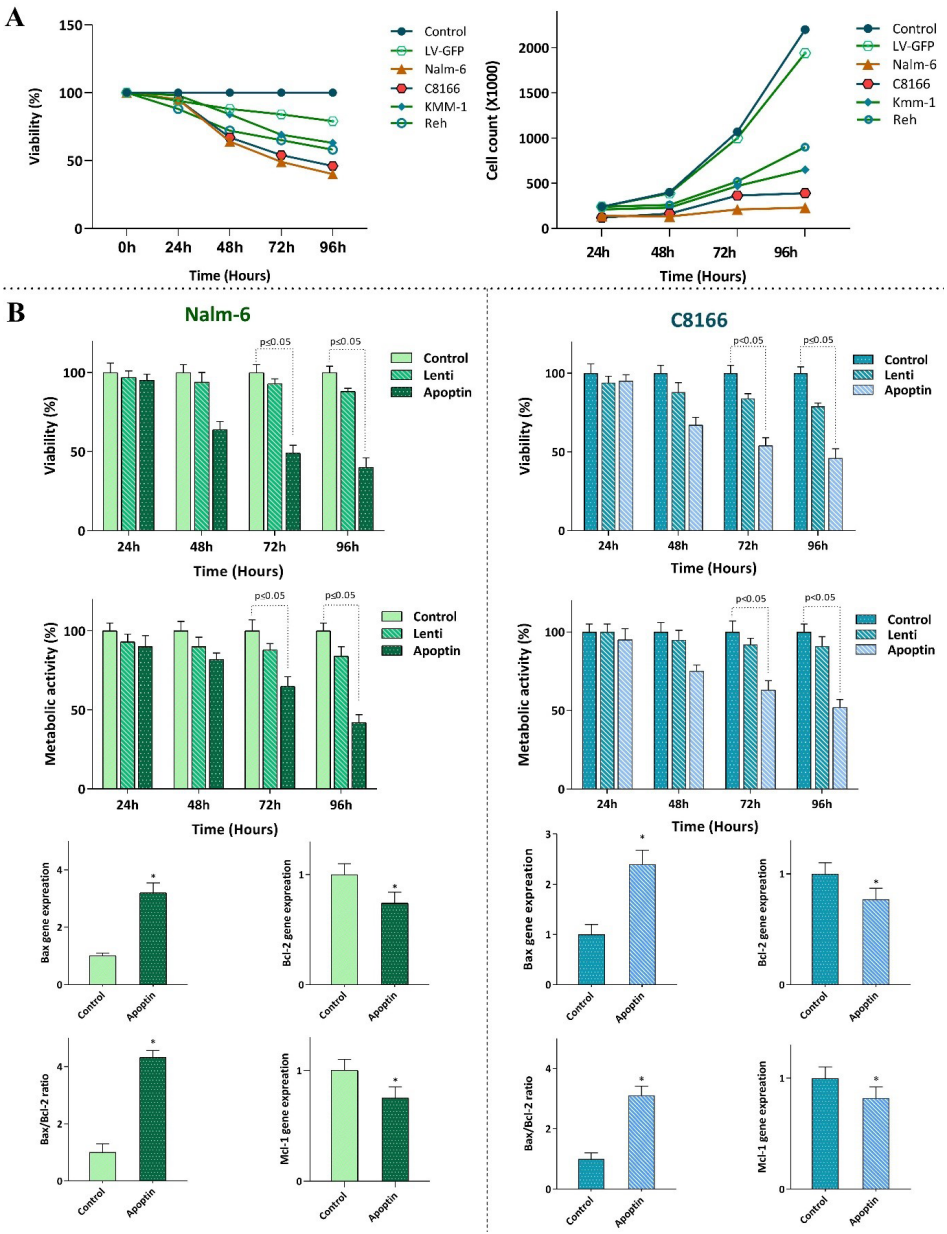
The effect of apoptin expression on the survival and proliferative capacity of leukemic cells.** A)** Transfection of apoptin gene into the leukemic cells was coupled with the reduction in the viability and the cell count of the cells. **B)** Transfection of Nalm-6 and C8166 cells with apoptin resulted in the reduction in the viability and metabolic activity of the cells probably through altering the balance between the expression levels of the pro- and anti-apoptotic target genes. Values are given as mean ± SD of three independent experiments. * *P* ≤ 0.05 represented significant changes from the control.

Having successfully transduced apoptin gene into the leukemic cells and evaluated its expression, it was of particular interest to ascertain whether the elevated expression of this gene diminished survival and proliferation of the leukemic cells. The results of trypan blue showed that apoptin expression potently decreased viability, and the number of viable cells of all the transduced cell lines models (Figure 2A); however, we observed a varied sensitivity pattern between the cells. Following 96 hours of treatment, both Nalm-6 and C8166 showed to be highly sensitive to apoptin expression as compared to other cells, and thereby, we selected these cell lines for further experiments. In the light of time-dependent anti-survival effects of this gene, as revealed by the results of both trypan blue and MTT assays (Figure 2B), and given to the crucial role of apoptin in the regulation of mitochondrial-dependent apoptosis in malignant cells,^9^ the impact of the over-expressed apoptin on induction of apoptotic cell death was investigated using qRT-PCR. Our results showed that upon apoptin transduction, there was an elevation in the expression level of Bax, while a significant decrease in the expression levels of MCL-1 and Bcl2 was detected in both Nalm-6 and C8166 cell lines (Figure 2B).

### Anti-proliferative effects of apoptin were mediated through induction of G2/M cell cycle arrest

**Figure 3:**
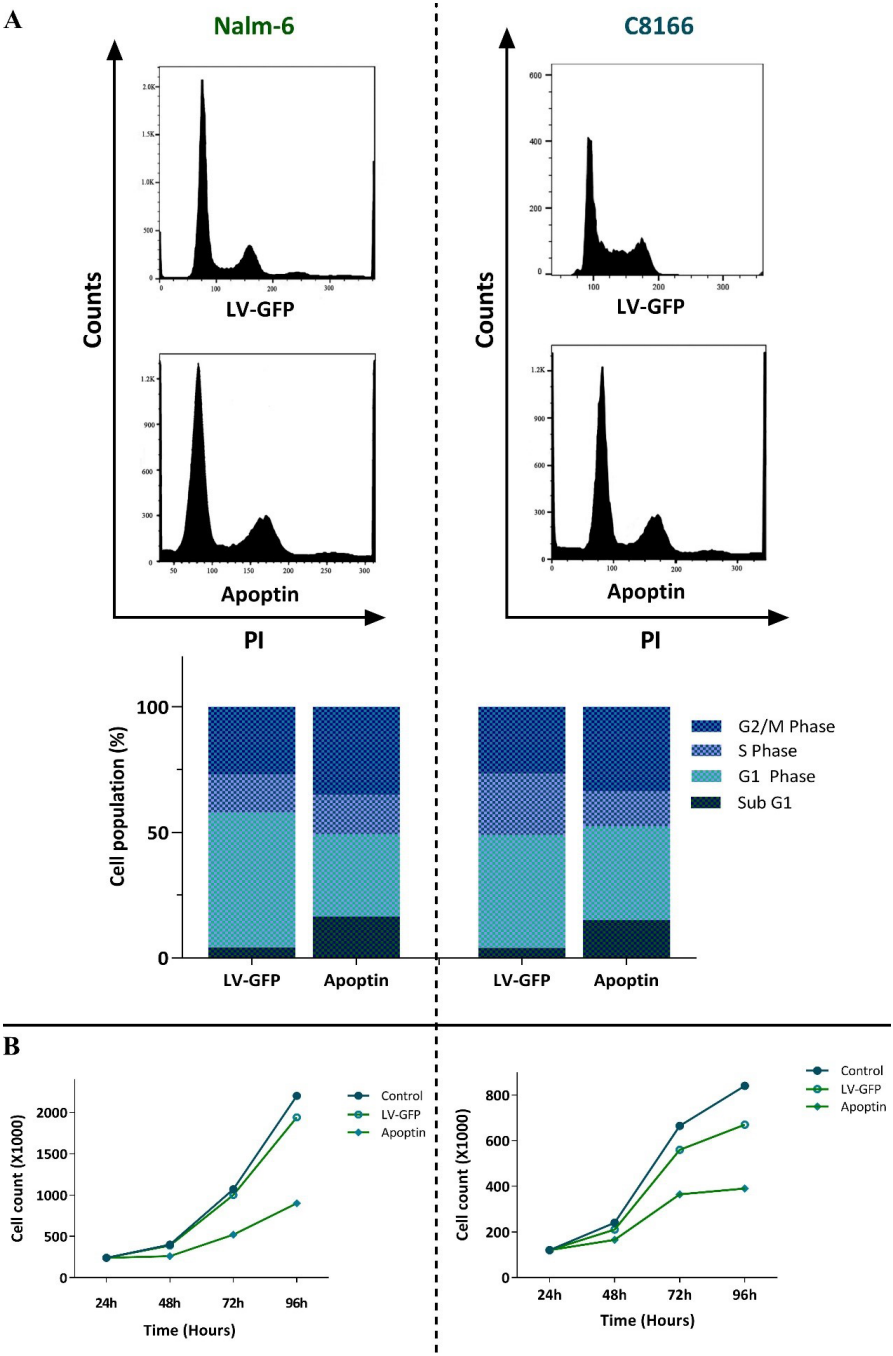
The effects of apoptin overexpression on the proliferative capacity of Nalm-6 and C8166 cells.** A)** Apoptin overexpression not only increased the percentage of the cells in the sub-G1 phase but also induced G2/M cell cycle arrest in both cell lines. **B)** Results of trypan blue exclusion assay revealed that the overexpression of apoptin in both cell lines diminished the number of viable cells. Values are given as mean ± SD of three independent experiments.

Previous studies showed that anti-cancer agents could induce their cytotoxic effects through either induction of apoptosis or halting the transition of the cells from different phases of the cell cycle.^10,11^ The impact of over-expressed apoptin on the regulation of apoptotic genes encouraged us to address whether the transduction of this gene could change the distribution of the cells in the sub-G1, a phase of the cell cycle indicative of the pro-apoptotic property of the cytotoxic agents. In agreement with the molecular analysis of apoptosis-related genes, we found that the overexpression of apoptin in both cell lines was coupled with the sensible blockage of the cells in the sub-G1 phase of the cell cycle (Figure 3A); shedding more light on the anti-survival efficacy of this gene. The impacts of apoptin on the progression of Nalm-6 and C8166 cell cycle were not restricted only to sub-G1, as the analysis of the DNA content showed that over-expressed apoptin was also able to augment the percentage of the cells in the G2/M phase (Figure 3A). The anti-proliferative property of apoptin, as revealed by the decreased cell population in the S phase, was further confirmed by the results of the trypan blue exclusion assay showing that both Nalm-6 and C8166 cells with over-expressed apoptin had a lower number of viable cells as compared with LV-GFP-infected cells (Figure 3B).

### Apoptin-overexpression enhanced the anti-leukemic effects of pan PI3K inhibitor BKM120

**Figure 4:**
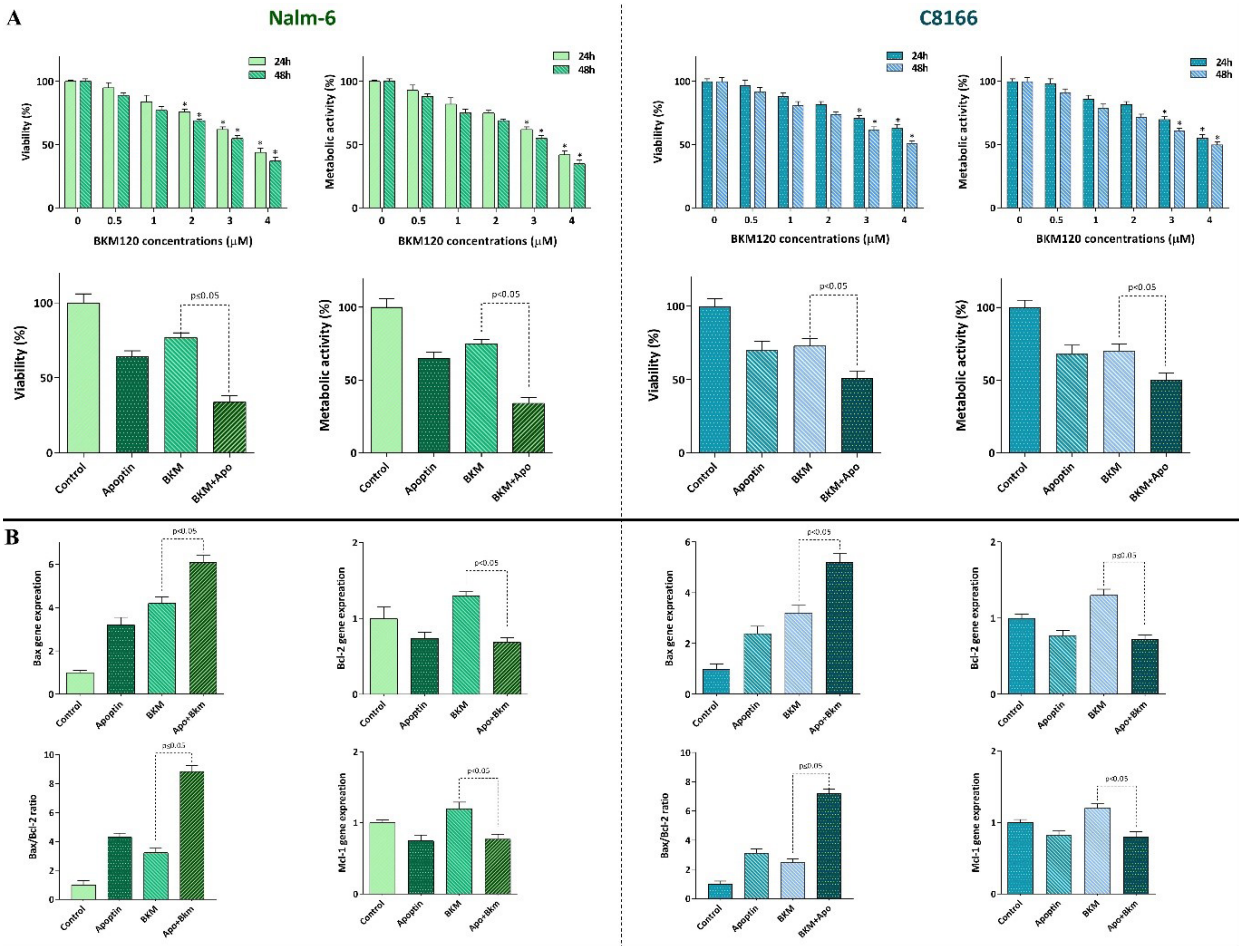
Overexpression of apoptin in both cell lines could potentiate the anti-leukemic property of pan PI3K inhibitor BKM120.** A** and **B)** Transduction of apoptin in both Nalm-6 and C8166 potentiated the anti-survival capacity of BKM120 possibly through altering the expression levels of both pro- and anti-apoptotic genes. Values are given as mean ± SD of three independent experiments. * *P* ≤ 0.05 represented significant changes from the control.

The PI3K signaling pathway has been suspected for the maintenance of cell survival in leukemic cells through regulation of a wide range of genes that participate in the regulation of apoptosis.^12^ Accordingly, when we treated Nalm-6 and C8166 with pan-PI3K inhibitor BKM120, we found that the inhibition of this axis was associated with the reduction of cell survival and proliferative capacity (Figure 4A). Notably, when apoptin gene was transfected in these cells, the ability of BKM120 to reduce the survival of the cells was significantly enhanced (Figure 4A). Our findings were further confirmed by qRT-PCR analysis showing that while single agent of BKM120 had a minimal impact on the expression levels of apoptotic-related genes, its effects on the expression levels of the indicated genes was potentiated more vigorously in the presence of apoptin overexpression (Figure 4B); suggesting that the overexpression of apoptin could be used alongside the PI3K inhibitors.

### Apoptin overexpression enhanced BKM120 effects on cell cycle progression in Nalm-6 and C8166

**Figure 5:**
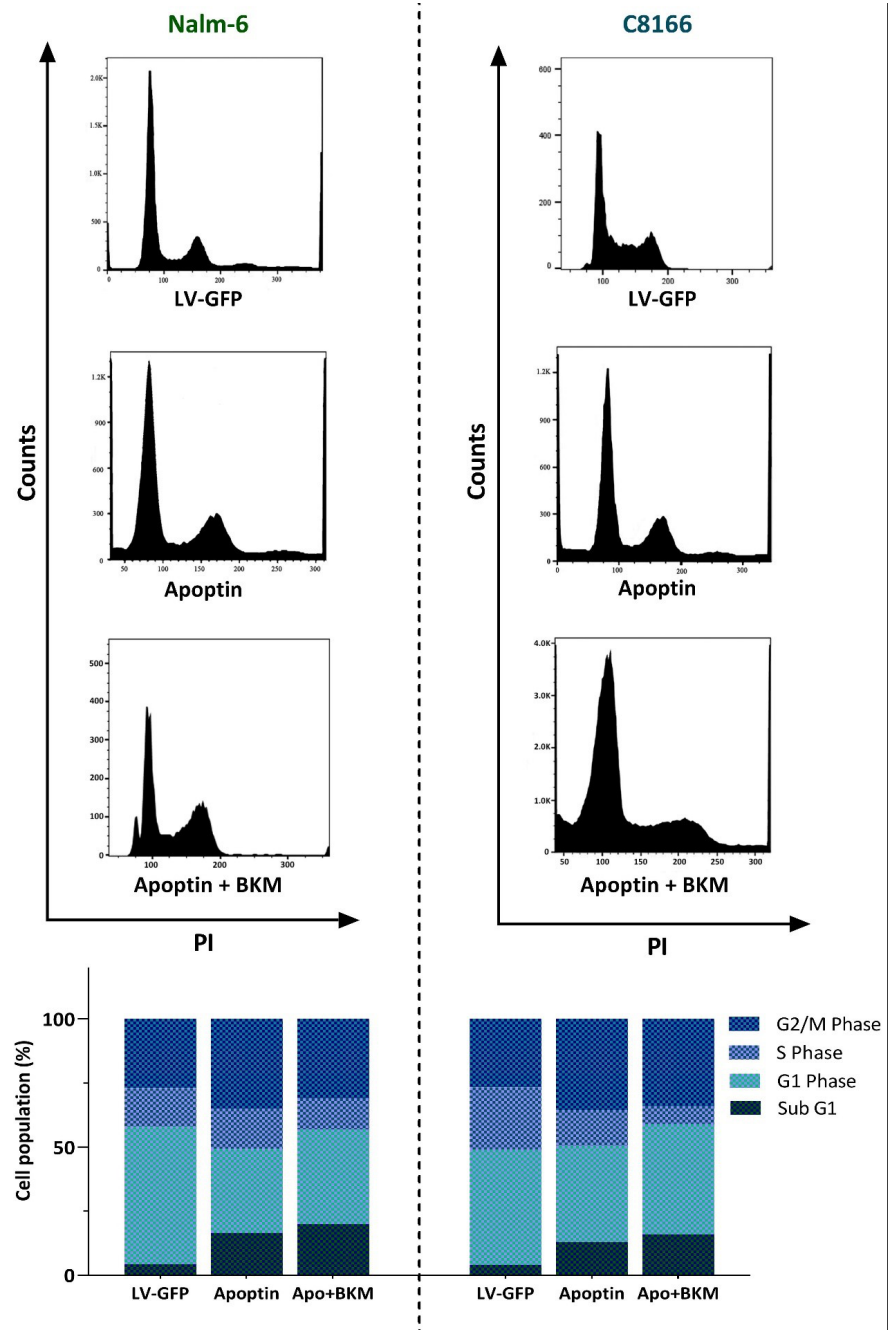
Evaluating the effects of apoptin on BKM120-induced anti-proliferative effects. The overexpression of apoptin could enhance the impact of BKM120 on the progression of the cell cycle. Upon expression of apoptin, BKM120 could more vigorously halt the progression of the cells from the G2/M phase of the cell cycle. Moreover, the percentage of the cells accumulated in the Sub-G1 phase also elevated in response to this combinational therapy.

To delve more into the mechanisms through which apoptin could increase the anti-leukemic property of PI3K inhibition, we aimed to address the behavior of apoptin-transduced cell lines in the presence of BKM120. Analysis of DNA content verified that upon overexpression of apoptin in both Nalm-6 and C8166, the ability of BKM120 in halting the progression of the cell cycle was potentiated. In agreement with the previous results, the distribution of the cells in the cell cycle showed that in apoptin-transduced cells BKM120 more vigorously induced G2/M cell cycle arrest and also remarkably reduced the number of cells in the S phase (Figure 5). Accordingly, investigating the percentage of the cells in Sub-G1 showed that the overexpression of apoptin together with the PI3K inhibition resulted in a more accumulation of the cells in this phase (Figure 5), which was in agreement with the results of qRT-PCR showing that the overexpression of apoptin in leukemic cell lines potentiated the impact of BKM120 on both pro- and anti-apoptotic target genes (Figure 4B).

## Discussion

Inducing apoptosis in cancer cells has long been considered to be an effective strategy to combat cancers and from the identification of apoptotic pathways, an ever-increasing attempt has been established to recruit this system in novel anti-cancer strategies.^13^ Indeed, numerous anti-cancer agents, either categorized as conventional or novel agents, debate their popularity due to their ability to trigger apoptotic cell death in cancer cells. However, the intelligent characteristic of malignant cells over this period of endless war has equipped them with the systems to evade the death signals and find a way to protect themselves from the devastating effects of these agents.^14,15^ One of the main mechanisms aiding malignant cells in this path is mediated by induction of anti-apoptotic genes to the extent that counteract with the effects of the death signals.^16^ The association of anti-apoptotic target genes in induction of resistance phenotype has been reported in several studies.^17^ This characteristic is now recruiting by the novel drug design to find a tactic to rebalance the expression of the anti- and pro-apoptotic genes in order to induce cell death in cancer cells.

One of the main mechanisms that could put a reality to this hypothesis is mediated through gene therapy, a technique that could integrate a desired gene into the genome of the malignant cells. apoptin, a protein that is derived from the chicken anemia virus, has been shown to possess cancer-specific cytotoxicity effects.^18-21^ In the present study, we aimed to investigate whether constructing a lentiviral vector encoding a green fluorescent protein-apoptin fusion gene (LV-GFP-AP) that can efficiently deliver apoptin into leukemic cells could be an effective strategy to reduce the survival of the neoplastic cells. Interestingly, we found that while overexpression of apoptin in a panel of leukemic cells significantly reduced the survival rate, LV-GFP (control vector) was unable to induce any significant cytotoxic effects on the indicated cell lines. Moreover, we found that the expression of apoptin in Nalm-6 and C8166 cells, which were more sensitive to the cytotoxic effects of this gene, not only induced a G2/M cell cycle arrest but also induced apoptotic cell death through alteration of apoptotic target genes. This finding was in harmony with the results of Li et al indicating that apoptin overexpression induces apoptosis in cancer cells via regulating a wide range of signaling pathways.^22^ In addition, there is another investigation suggesting that apoptin could induce Bcl2-stimulated apoptosis in various human tumor cells.^23^

**Figure 6:**
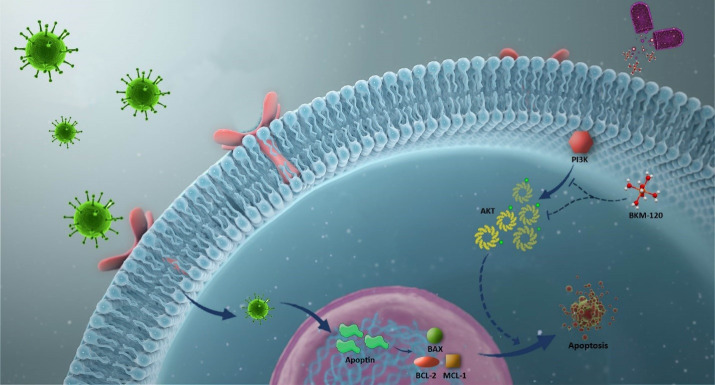
Schematic representation proposed for the plausible mechanism by which overexpression of apoptin could enhance the anti-leukemic effect of PI3K inhibitor.When leukemic cells were subjected to the lentivirus containing the apoptin gene, both the viability and the proliferative capacity of the cells were hampered as a result of the induction of cell death. The overexpression of apoptin in leukemic cells could also potentiate the anti-leukemic property of BKM120, a well-known PI3K inhibitor, upon altering the balance between the expression levels of pro- and anti-apoptotic target genes.

Previous studies showed that the aberrant activation of the PI3K signaling pathway in most of the hematologic malignant cells is associated with the maintenance of leukemic cells survival and proliferation.^24,25^ Accordingly, it has been widely shown that when this axis is blocked in leukemic cells, the ability of the cells to survive and proliferate would be diminished significantly.^26-28^ However, despite a favorable anti-leukemic effect, one reason that restricts the application of the PI3K inhibitors in human leukemia is due to their short-term response given to the acquisition of resistance. BKM120, a well-known pan-PI3K inhibitor, has shown to have promising anti-leukemic effects in a wide range of hematologic malignant cell lines.^29-32^ The results of the previous study indicated that as compared to acute myeloid leukemia cell lines, the survival of pre-B ALL-derived Nalm-6 cells reduced at the higher concentrations of the inhibitor.^33^ Several mechanisms have been proposed for this differential response; however, the precise mechanism responsible for this phenomenon has not yet been elucidated. To the best of our knowledge, no study has addressed the effect of apoptin overexpression on the anti-leukemic properties of BKM120 and we reported for the first time that apoptin overexpression in Nalm-6 cells could remarkably robust the anti-tumor activity of BKM120, while reduced its concentrations (Figure 6). This finding, which was further confirmed in another cell line, suggested that probably the overexpression of apoptosis-related genes could be a probable mechanism through which the resistance to PI3K inhibitors could be bypassed. Moreover, we found that through overexpression of apoptin, the ability of the lower concentration of BKM120 (2 µM) was significantly increased to halt the transition of the cells from Sub-G1 phase of the cell cycle, at least partly, through up-regulation of Bax.

## Conclusion

Taken together, the results of the present study shed light on the promising effect of apoptin overexpression in leukemic cells and suggested that transduction of this gene into leukemic cells not only induced G2/M cell cycle arrest but also induced apoptotic cell death by altering the balance between pro- and anti-apoptotic target genes. Of particular interest, the efficacy of apoptin transduction was not limited to these findings as we reported for the first time that the overexpression of this gene could potentiate the anti-leukemic property of pan PI3K inhibitor BKM120 in lymphoblastic leukemia cell lines; shedding light on the possibility of this approach in the therapeutic strategies of ALL.

## Acknowledgments

The authors would like to express their gratitude to Shahid Beheshti University of Medical Sciences (Tehran, Iran) for supporting this study (Grant number: 22798)

## Ethical issues

This study does not contain human participants or animals, and it was approved by the Shahid Beheshti University of Medical Sciences Ethics Committee (IR.SBMU.RETECH.REC.1399.303).

## Conflict of interest

The authors declare that they have no conflict of interest.
